# Data set and methodology involving pedagogical approaches to teach mental health and substance use in dental education

**DOI:** 10.1186/s13104-022-05960-1

**Published:** 2022-02-19

**Authors:** Mario Brondani, Rana Alan, Leeann Donnelly

**Affiliations:** 1 Department of Oral Health Science, Faculty of Dentistry, University of British Columbia, 2199 Wesbrook Mall, Vancouver, BC V6T 1Z3 Canada; 2 Private Practice Dentist. Smile Dental Center, East Boston, USA; 3 Department of Oral Biological & Medical Sciences, Faculty of Dentistry, University of British Columbia, Vancouver, Canada

**Keywords:** Secondary data analysis, Reflections, Substance use, Mental health disorders, Undergraduate education, Vignette

## Abstract

**Objective:**

In this Data note, we provide a raw data set in the form of brief self-guided reflections. We also present the methodological approach to generate these reflections including an educational vignette so that other dental schools can plan for their teaching activities involving mental health and substance use topics.

**Data description:**

Between 2015/16 and 2018/19, the University of British Columbia’s (UBC) undergraduate dental and dental hygiene students submitted optional written guided reflections to address ‘*how can an educational vignette, depicting a patient with a history of substance use and mental health disorders accessing dental care, promote an open dialogue about stigma?*’ From a total of 323 undergraduate students, 148 anonymous reflections between 200 and 400 characters each were received. The main ideas that may emerge from the reflections include ‘exploring power relations’ and ‘patient-centered care approach to counteract stigma’.

## Objectives

The objective of this data note is twofold: (1) to illustrate the methodological approach used to generate guided reflections at undergraduate level aided by a patient-based vignette portraying an individual with a history of substance use and mental health disorders; and (2) to provide a summary of the raw data set in the form brief educational reflections submitted anonymously by undergraduate dental and dental hygiene students. These reflections were used in our recent publication titled.The role of an educational vignette to teach dental students on issues of substance use and mental health disorders at the University of British Columbia: An exploratory Qualitative study [1]. By offering the reader with a *road map* to generate such reflections, and a summary of the reflections themselves, we hope to engage other dental schools in planning their educational teaching activities on issues pertaining to mental health and substance use for dental and dental hygiene students as we have advocated over the years [2–4]

## Data description

Secondary analysis is common with qualitative—numerical—data in which the original data set [5] is re-analyzed for new insights [6]. Less frequent is the re-evaluation of quantitative—textual—data given their inherent subjective nature [7]. However, an original compilation of textual data [8] can be re-interpreted to unravel new understandings [9]. And that is what we hope to accomplish with this Data note. Between 2015/16 and 2018/19 [10], we have asked undergraduate dental students at the UBC to reflect on issues of stigma and discrimination towards substance use and mental health disorders as a socially responsible provider [11–13]. These anonymous reflections were prompted by an educational vignette posed during a 2.5 h didactic session [3]—please see a suggested methodological approach to generate reflection data [14].

We have gathered 148 anonymous reflections ranging from 202 to 405 characters each, leading to more than 284 double-spaced pages of text. We present the reflection data according to the five major ideas that might or might not coincide with the readers’ take on the raw data:

### Vignettes in undergraduate education

This file may attest for the value of a vignette to generate discussion within an undergraduate classroom. The reader may also appreciate how vignettes present themselves as an alternative way to facilitate the discussion of sensitive issues that emerge from lack of knowledge and awareness [15].

### Vignette to explore stigma and mental health disorders

In this file, the reader can understand mental health disorders as the most often stigmatized conditions [16, 17]. However, issues of stigma based on other characteristics or traits may also surface [18–22].

### The interplay of Link & Phelan’s stigma framework

This file shows how the vignette helped students to discuss Link & Phelan’s framework on stigma [23]. The reader might explore how labels become the basis for stereotypes as they set into action negative images about certain conditions and individuals [24, 25]

### Patient-centered care approach to counteract stigma

The textual data in this file may counteract stereotypes and labels about certain patients once health professionals consider patients’ history and environmental, societal and personal factors. Such consideration falls within a patient-centred care approach [26, 27]

### Not a one-size-fits-all use of the vignette

This file gives the reader the opportunity to explore how the vignette might have not engaged students into the discussions [8]. It might also point out to the fact the vignette may not have offered sufficient information about the patient [28, 29].

## Limitations

- The reflection data came from a study involving a small number of undergraduate students from one Canadian dental school only, albeit involving cohorts from multiples years.
- The method for gathering the reflection was a one-time invitation per academic year.
- We could not gather the information pertaining to student’s previous substance use os stigma reduction training.
- These cohorts might not typically represent all oral health care professionals; generalizability of the reflections is limited.
- The optional nature of the reflections might have limited the amount of data, but might have engaged those students who had something to say.
- Although we gathered 148 reflections, ideas were repeated within many submissions over the years.
- The session described herein was modified during the Covid-19 pandemic in 2019/20 and 202/21, where no reflections were gathered.

Table 1 provides an overview of all data set described in this Data note.

**Table 1:** Data set description as presented in this Data note

Label	Name of data file/data set	File types (file extension)	Data repository and identifier (DOI or accession number)
Data file 1	Methodological_approach_to_generate_reflection_and_reflective_notes	Microsoft Word (.docx)	https://doi.org/10.5061/dryad.gtht76hmv [14]

## Data Availability

The data described in this Data note can be freely and openly accessed on Dryad under Ref. [14]. Please see references [1, 3, 25] for details to already analyzed data.

## Abbreviations

COVID: coronavirus disease
Q&A: Question and answer
UBC: University of British Columbia

## References

[1] Brondani M, Alan R, Donnelly L (2021). The role of an educational vignette to teach dental students on issues of substance use and mental health disorders in patients at the University of British Columbia: an exploratory Qualitative study. BMC Med Educ.

[2] Brondani M, Patanaporn K (2013). Integrating issues of substance abuse and addiction into the predoctoral dental curriculum. J Dent Educ.

[3] Brondani M, Alan R, Donnelly L (2017). Stigma of addiction and mental illness in healthcare: the case of patients’ experiences in dental settings. Plos ONE.

[4] Brondani M, Park PE (2011). Methadone and oral health—a brief review. J Dent Hygiene.

[5] Brondani MA, Cruz-Cabrera MA, Colombe C (2010). Oral sex and oral cancer in the context of human papillomavirus infection: lay public understanding. Oncol Rev.

[6] Brondani M, Siqueira AB, Alves CMC (2019). Exploring the lay public and dental professional knowledge around HPV transmission via oral sex and oral cancer development. BMC Public Health.

[7] Smith J, Firth J (2011). Qualitative data analysis: the framework approach. Nurse Res.

[8] Brondani MA, MacEntee MI, Bryant SR, O’Neill B (2008). Using written vignettes in focus groups among older adults to discuss oral health as a sensitive topic. Qual Health Res.

[9] Brondani M (2010). The voice of the elderly in accepting alternative perspectives on oral health. J Comm Dent Health.

[10] Brondani MA, Clark C, Rossoff L, Aleksejuniene J (2008). An evolving community-based dental course on professionalism and community service. J Dent Educ.

[11] Mariño R, Hawthorne L, Morgan M, Ismail A (2012). Transcultural skills content in a dental curriculum: a comparative study. Eur J Dent Educ.

[12] Brondani MA (2012). Teaching social responsibility through community service-learning in predoctoral dental education. J Dent Educ.

[13] Brondani M, Harjani M, Siarkowski M, Adeniyi A, Butler K, Patel K, Dakelth S, Maynard R, Ross K, Donnelly L (2020). The community as the teacher in dental education on issues of social responsibility, substance use and queer health. Plos ONE.

[14] Brondani M. Methodological approach to generate reflection and reflective notes, Dryad, Dataset. 2021. 10.5061/dryad.gtht76hmv.

[15] Jorm AF, Reavley NJ, Ross AM (2012). Belief in the dangerousness of people with mental disorders: a review. Aust N Z J Psychiatry.

[16] Corrigan PW, Watson A (2002). Understanding the impact of stigma on people with mental illness. World Psychiatry.

[17] Quinn N, Knifton L (2014). Beliefs, stigma and discrimination associated with mental health problems in Uganda: implications for theory and practice. Int J Soc Psychiatry.

[18] DePierre JA, Puhl RM, Luedicke J (2013). A new stigmatized identity? Comparisons of a “food addict” label with other stigmatized health conditions. Basic Appl Soc Psychol.

[19] Brondani M, Donnelly L, Postnikoff J (2016). “I’m not HIV positive, I’m undetectable”: community forum on issues of stigma. Stigma Health.

[20] Brondani M, Morini N, Kerston RP (2012). Community-based research among marginalized HIV populations: issues of support, resources and empowerment. Interd Perspect Inf Dis.

[21] Mago A, Brondani M, MacEntee M, Frankish J (2018). The angry voices of homeless adults in Vancouver’s downtown east-side. Commun Dent Oral Epidemiol.

[22] Brondani M, Donnelly L (2021). The HIV and SARS-CoV-2 parallel in dentistry from the perspectives of the oral health care team. J Dent Res Clin Transl Res.

[23] Link BG, Phelan JC (2001). Conceptualizing stigma. Ann Rev Sociol.

[24] Najman JM, Klein D, Munro C (1982). Patients characteristics negatively stereotypes by doctors. Soc Sci Med.

[25] Alan R. Stigma of addiction and mental health in dental settings: patients' experiences. MSc thesis. 2014. https://open.library.ubc.ca/cIRcle/collections/ubctheses/24/items/1.0167504 Accessed 29 May 2021.

[26] Bedos C, Loignon C (2011). Patient-centred approaches: new models for new challenges. J Can Dent Assoc.

[27] Apelian N, Vergnes JN, Hovey R, Bedos C (2017). How can we provide person-centred dental care?. Br Dent J.

[28] Freeman R, Ismail A (2009). Assessing patients' health behaviours. Essential steps for motivating patients to adopt and maintain behaviours conducive to oral health. Monogr Oral Sci.

[29] Rajabiun S, Fox JE, McCluskey A (2012). Patient perspectives on improving oral health-care practices among people living with HIV/AIDS. Public Health Rep.

